# The atypical RhoGTPase RhoE/Rnd3 is a key molecule to acquire a neuroprotective phenotype in microglia

**DOI:** 10.1186/s12974-018-1386-z

**Published:** 2018-12-15

**Authors:** Veronika E. Neubrand, Irene Forte-Lago, Marta Caro, Mario Delgado

**Affiliations:** Instituto de Parasitología y Biomedicina López-Neyra, IPBLN-CSIC, Avd. Conocimiento 17, PTS Granada, 18016 Granada, Spain

**Keywords:** Microglia, Neuroinflammation, siRNA screen, Cell morphology

## Abstract

**Background:**

Over-activated microglia play a central role during neuroinflammation, leading to neuronal cell death and neurodegeneration. Reversion of over-activated to neuroprotective microglia phenotype could regenerate a healthy CNS-supporting microglia environment. Our aim was to identify a dataset of intracellular molecules in primary microglia that play a role in the transition of microglia to a ramified, neuroprotective phenotype.

**Methods:**

We exploited the anti-inflammatory and neuroprotective properties of conditioned medium of adipose-derived mesenchymal stem cells (CM) as a tool to generate the neuroprotective phenotype of microglia in vitro, and we set up a microscopy-based siRNA screen to identify its hits by cell morphology.

**Results:**

We initially assayed an array of 157 siRNAs against genes that codify proteins and factors of cytoskeleton and activation/inflammatory pathways in microglia. From them, 45 siRNAs significantly inhibited the CM-induced transition from a neurotoxic to a neuroprotective phenotype of microglia, and 50 siRNAs had the opposite effect. As a proof-of-concept, ten of these targets were validated with individual siRNAs and by downregulation of protein expression. This validation step resulted essential, because three of the potential targets were false positives. The seven validated targets were assayed in a functional screen that revealed that the atypical RhoGTPase RhoE/Rnd3 is necessary for BDNF expression and plays an essential role in controlling microglial migration.

**Conclusions:**

Besides the identification of RhoE/Rnd3 as a novel inducer of a potential neuroprotective phenotype in microglia, we propose a list of potential targets to be further confirmed with selective activators or inhibitors.

**Electronic supplementary material:**

The online version of this article (10.1186/s12974-018-1386-z) contains supplementary material, which is available to authorized users.

## Background

Neuroinflammation is a fundamental process contributing to the death of neurons in neurodegerenative diseases, for example, in Parkinson’s Disease (PD) [1], Alzheimer’s Disease, and Multiple Sclerosis [2]. The underlying neuroinflammation in these neurodegenerative diseases are characterized by microglia activation [2, 3]. During this inflammatory process, microglia acquire an amoeboid cell shape [4], and amongst other mechanisms, they decrease the release of neurotrophic factors, such as the brain-derived neurotrophic factor (BDNF), and secrete cytotoxic substances, which lead to neuronal death [5]. In contrast, in a healthy brain, microglia are found in a ramified morphology and support a variety of central nervous system (CNS) functions by secreting neurotrophic factors. Interestingly, microglia are not permanent in one or another activity state, but are rather able to switch between different phenotypes and activity states [6]. Therefore, understanding the molecular machinery that reverses the inflammatory activation of microglia is essential to protect from neurodegeneration. Hence, to identify the intracellular players of this reversion in a systematic manner, we combined two experimental approaches in this study.

Firstly, our previous work indicates that adipose-derived mesenchymal stem cells (ASCs) exert important anti-inflammatory actions on microglia. We observed that primary microglia exposed to ASCs or their secreted factors (conditioned medium, CM) underwent a dramatic cell shape change into a highly elongated morphology in vitro [7], similar to the phenotype of microglia observed in a healthy brain [8]. The elongation induced by CM was associated with an upregulation of neurotrophic factors, such as BDNF, which is indicative of the acquisition of a neuroprotective phenotype [7]. Thus, CM-stimulated microglia represent an ideal tool to study the intracellular events necessary for the transition from inflammatory activated to non-inflammatory neuroprotective microglia. Indeed, we previously identified that the small RhoGTPases Rac1 and Cdc42, which are important regulators of the actin cytoskeleton [9, 10], play major roles in this phenotypic transition [7].

Secondly, we present here our novel results of a small interference RNA (siRNA) screen targeting a panel of cytoskeletal and inflammatory genes in primary microglia. For our siRNA screen, we chose a microscopy-based screen as readout, since the phenotype transition of microglia is easily detectable by light microscopy and its outcome is directly visible. Although RNAi screens have been applied to create big datasets of image-based analysis of cell lines in high-throughput format [11, 12], in the case of microglia available, cell lines do not always resemble the physiology and morphology of primary microglia [13] and our own experience. Therefore, despite the technical challenge associated with the use of primary cells, we chose primary murine microglia as our model system. We were able to develop a reliable protocol to transfect primary microglia with a panel of siRNA, and optimized the experimental assay for its reproducible readout. Then, we exploited the anti-inflammatory properties of CM on microglia [7], as described above, in combination with the siRNA screen, and in this way, we identified a list of molecules that were implicated in the reversion from activated to neuroprotective microglia. From this list, we validated seven candidates, of which three of them also downregulated BDNF expression and other three molecules enhanced microglia migration. We found that the atypical RhoGTPase RhoE/Rnd3 was a common hit in both secondary screens enabling us to assign a novel function as a potential regulator of a neuroprotective microglia phenotype to this molecule.

## Methods

### Animals and ethical statement

Female C57Bl/6 mice (7–8  weeks old) were purchased from Charles River. They were used for obtaining P0 to P2 newborns to isolate primary microglia and for obtaining fat tissue to isolate ASCs. Mice were housed in a controlled-temperature/humidity environment (22 ± 1 °C, 60–70% relative humidity) in individual cages (10 mice per cage, with wood shaving bedding and nesting material), with a 12-h light/dark cycle (lights on at 7:00 a.m.) and fed with rodent chow (GlobalDiet 2018, Harlan) and tap water ad libitum. The experimental protocols of this study conform to EU Directive 2010/63 and followed the ethical guidelines for investigations with experimental animals approved by the Ethics Review Committee for Animal Experimentation of Spanish Council of Scientific Research. Animal studies are reported in compliance with the ARRIVE guidelines [14].

### Cell isolation and cultures

Primary microglia were prepared from P0 to P2 newborn C57Bl/6 mice, as described previously [7]. Briefly, dissected brains were discarded of olfactory bulb, cerebellum, hindbrain, and meninges. Then, they were homogenized in microglia growth medium consisting in DMEM (Invitrogen), supplemented with 10% fetal bovine serum (FBS, Gibco), 10% horse serum, and 1% penicillin/streptomycin (Gibco), using a Pasteur pipette and a 23-G syringe. Brain homogenates were centrifuged. The resulting cells were plated in poly-d-lysine-coated flasks and incubated with microglia growth medium for 10–12 days at 37 °C and 5% CO_2_. Microglia were harvested on a shaker for 2 h at 200 rpm and plated on poly-d-lysine-coated cover slips at a density of 12,000 cells/cm^2^ or tissue culture 6-well plates in microglia growth medium at a density of 37,000 cells/cm^2^. After 24 h, microglia growth medium was replaced by fresh microglia growth medium.

ASCs were isolated from adipose tissue of adult C57Bl/6 mice as previously described [15]. These cells showed a fibroblast-like morphology and differentiation capacity to the adipocytic and osteocytic lineages and expressed the phenotype MHC^−^II^−^CD14^−^CD18^−^CD31^−^CD34^−^CD45^−^CD80^−^CD117^−^CD144^−^CD13^+^CD44^+^CD29^+^CD54^+^CD73^+^CD90^+^CD105^+^CD106^+^CD166^+^. CM was collected from passage 2 until passage 6 of ASC cultures, which were plated at a cell density of 15,000 cells/cm^2^, grown for 2 days before collecting their supernatant and then stored at − 20 °C. Before use, CM was quickly thawed and passed through a 0.2-μm filter.

### siRNA transfection

A custom-made siRNA library against 157 target genes (with three individual siRNAs against each gene) was ordered from Life Technologies. Primary microglia were plated for 48 h as described above. Then, the growth medium was replaced and cells grown on cover slips in 48-well plates were transfected with 12.5 pmol siRNA and 0.375 μl of Lipofectamine 3000 (Invitrogen) according to the manufacturer’s instructions. For 24-, 12-, or 6-well plate cultures, reagents were proportionally up-scaled. Four days after the siRNA transfection, cells were treated with CM for 4 h and fixed to determine their cell shape. Alternatively, after 4 days, cells were harvested for Western blot or RT-qPCR analysis. Not transfected cells or cells that were transfected with a control Scrambled siRNA were used as reference in all studies.

### Determination of cell shape and circularity

Microglia plated on cover slips were fixed for 4 min in ice-cold methanol and stained with Isolectin B4, labeled with AlexaFluor 568 (Invitrogen) for 30 min at room temperature and washed two times with PBS. Images were acquired with a × 10 objective on an Olympus IX 81 fluorescence microscope. To determine the circularity or “form factor” [7, 16], photos of three fields of view were taken per siRNA and analyzed by a Fiji/ImageJ macro to determine the circularity of each individual cell. Briefly, each image was processed by the median filter at a radius of 8 pixels, then a black and white threshold image was generated, cell surroundings were drawn, and the circularity within shape descriptions was determined as 4π*area/(perimeter)^2^. Cells touching the borders of the image were excluded from the quantification procedure. In total, the siRNA screen was performed three times so that the effect of each target gene on circularity was calculated from three independent experiments. Subsequently, the circularity values were normalized within each experiment and compared to microglia transfected with Scrambled siRNA. The fold change difference between the circularity value for each gene and the Scrambled siRNA was called the differential circularity, since it indicates the difference between the Scrambled siRNA and gene siRNA, and is given as logarithm (log_2_).

### Scratch assay

Migration of microglia was determined using a scratch assay. Cells were plated at 18,000 cells/cm^2^ and transfected as described above. Growth medium was replaced 3 days after transfection to CM, and the cover slip was scratched with a yellow pipette tip. Bright field images were taken 1 and 24 h after the scratch. The scratch width was analyzed with the Fiji/ImageJ macro Wound healing tool (MRI_Wound_Healing_Tool.ijm). The scratch width at 24 h was expressed as a percentage of the original scratch width at 1 h. For the normalization, the scratch width of the Scrambled siRNA-transfected cells was set to 1.0 in each experiment, and the scratch width of the siRNA-transfected cells were set to values proportional to 1.0.

### Western blot analysis

Microglia were transfected as described above and then harvested with cold lysis buffer consisting in 10 mM Tris-HCl pH 8.0, 150 mM NaCl, 1% Nonidet-P40, 1 mM EDTA, 10 mMNaF, 1 mM Na_3_VO_4_, and a cocktail of commercially available protease inhibitors (Sigma, containing 104 mM AEBSF, 80 μM Aprotinin, 4 mM Bestatin, 1.4 mM E-64, 2 mM Leupeptin, 1.5 mM Pepstatin A). After centrifugation for 15 min at 14,000 rpm, the protein concentration of the supernatants was determined by Bradford assay (Bio-Rad), and samples were prepared for SDS-PAGE with Laemmli SDS sample buffer. After semi-dry blotting or Western blotting in a wet chamber for Tiam1, PVDF membranes were blocked with 5% milk in TBS-Tween (0.1%) and incubated 8 h at 4 °C with the primary antibodies, rabbit anti-Ahrgef4, (Antibodies-Online), rabbit anti-IκBα (Cell Signaling), mouse anti-Rac1 (BD Transduction laboratories), rabbit anti-GAPDH (Sigma), mouse anti-Tiam1, and mouse anti-Map3k2 (both from Santa Cruz) diluted in 2% BSA/TBS-Tween (0.1%) or incubated 1 h at RT with the primary antibodies mouse anti-Creb1, rabbit anti-RhoE, rabbit anti-GM-CSFR, and rabbit anti-Mapk11 (all from Antibodies-Online) diluted in the blocking solution. Horseradish peroxidase-conjugated secondary antibodies (DakoCytomation) and ECL (GE Biotech) were used for detection. If necessary, membranes were stripped with stripping buffer (100 mM β-mercaptoethanol, 2% SDS, 62.5 mMTris pH 6.8) for 30 min at 55 °C.

### RNA extraction and RT-qPCR

Total RNA was extracted using Tripure (Roche) from microglia plated in 6-well plates. After DNase I treatment (Sigma), RNA (1 μg/sample) was reverse transcribed using RevertAid First Strand cDNA Synthesis kit (Fermentas) and random hexamer primers. The cDNA was analyzed by qPCR in triplicates on a Cfx96-Cycler (Bio-Rad) with the SensiFAST™ SYBR® No-ROX Kit (Bioline) and 2.5 pmol of the following primers: mouse Rac1 forward, CCC AAT ACT CCT ATC ATC CTC G; mouse Rac1 reverse, CAG CAG GCA TTT TCT CTT CC; mouse BDNF forward, CCC TCC CCC TTT TAA CTG AA; mouse BDNF reverse, GCC TTC ATG CAA CCG AAG TA with a primer efficiency of 100.7% and GAPDH forward and reverse primers from the RevertAid First Strand cDNA Synthesis kit (Fermentas), with a primer efficiency of 86.3%. After 42 cycles, the Ct values were determined. To normalize the samples, ΔCt between the gene of interest and GAPDH Ct values as reference gene was calculated. The *x*-fold difference in expression between the different treatments was then determined by subtraction of the ΔCt values and called ΔΔCt. Finally, the total change was calculated as 2^−ΔΔct^ and the relative amount compared to Scrambled siRNA-transfected cells was deducted.

### Elisa

BDNF protein levels in culture supernatants of siRNA-transfected primary microglia were determined using the Quantikine® ELISA Kit (R+D Systems®), a quantitative sandwich enzyme immunoassay, according to the manufacture’s recommendations.

### Statistical analysis

All data are expressed as the mean ± SEM. The data and statistical analysis comply with the recommendations on experimental design and analysis in pharmacology (Curtis et al., 2018). Statistical analysis was carried out with two-way ANOVA followed by Student’s *t* test. We assumed significance at *p* < 0.05. For the statistical analysis of the siRNA screen, cell HTS2 package version 2.38.0 [17] from R version 3.3.2 was applied to calculate and normalize all circularity values within each experiment, as it is the case in the normalization by experiments. Finally, these normalized values were compared for each gene to a Scrambled siRNA used as negative control sample using limma software version 3.30.11 [18]. Achieved results are presented as a “fold change” in the circularity, given as logarithm (log_2_) value. The threshold value between significant and not significant genes was determined by an adjusted *P* value: the Fold Discovery Rate (FDR). We applied the standardized FDR, as previously described [19].

## Results

### Rac1 siRNA efficiently inhibited CM-induced ramification

**Fig. 1:**
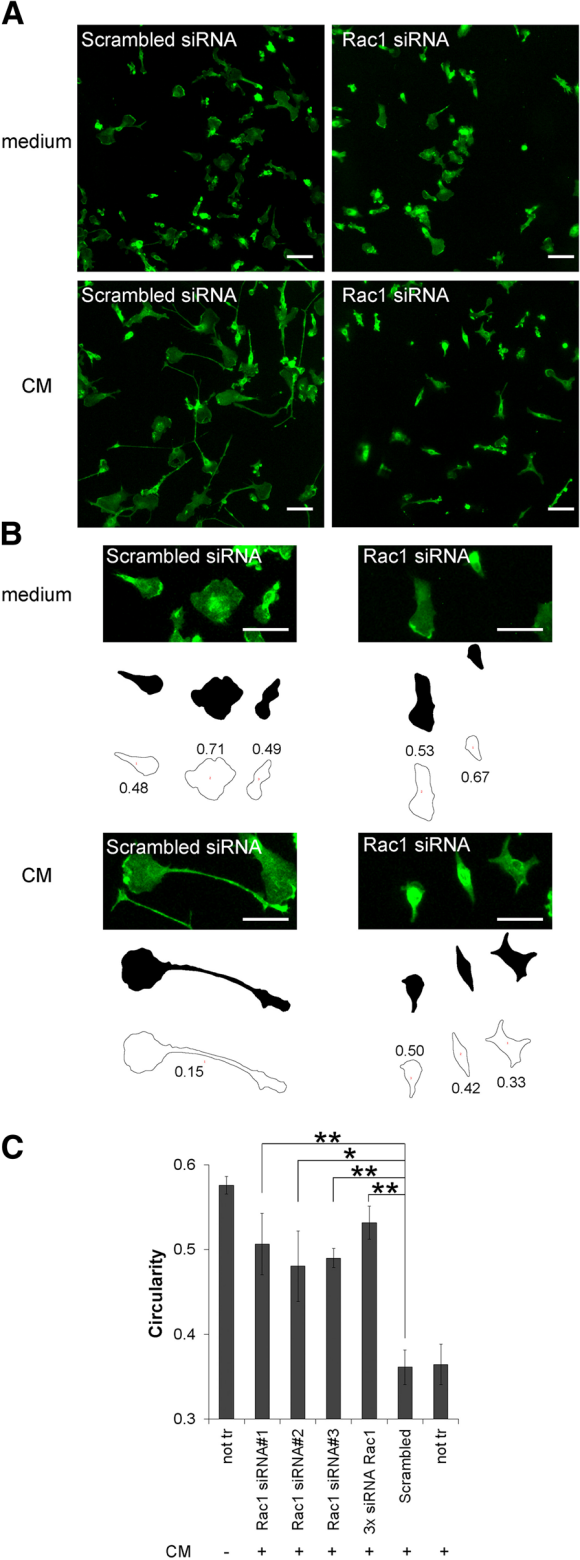
Rac1 siRNA efficiently inhibited CM-induced ramification. **a** Primary mouse microglia were transfected with Rac1 siRNAs or Scrambled siRNA (used as control) for 4 days, then incubated in normal medium or CM for 4 h and stained with the microglia surface marker isolectin B4 (green fluorescence). Microglia morphology was assessed on a fluorescence microscope. Scale bars, 20 μm. **b** High magnification of cells shown in **a** and the typical black and white threshold images of the cells generated by image analysis program Fiji/ImageJ are shown as an example. The outlines of each cell were drawn to measure cell perimeter and area, which are the parameters needed to calculate the circularity values (correspond to numbers near each outline). Note that cells touching the image edges were automatically excluded from the measurement. Scale bars, 20 μm. **c** Microglia were transfected with three individual siRNA oligonucleotides targeting Rac1 or a pool of them (3x siRNA Rac1) and then incubated with CM. Isolectin B4-stained microglia were visualized by microscopy, and the cell shape was quantified using the circularity value. Non-transfected cells (not tr) or cells transfected with Scrambled siRNA were used as controls of reference. Data are mean ± SEM of three independent experiments. *p < 0.05, **p < 0.01 vs. Scrambled siRNA

First of all, we established the experimental conditions to evaluate the efficiency of the siRNA knockdown in primary mouse microglia. Since a dominant-negative mutant of the small RhoGTPase Rac1 inhibited the CM-induced conversion to an elongated neuroprotective microglia [7], we expected the same phenotype for its siRNA knock down. Therefore, we used Rac1 as test protein. Indeed, Rac1 protein and mRNA expression were efficiently downregulated (66.1 ± 9.0% protein and 59.7 ± 5.1% mRNA at 72 h) after transfection with Silencer-Select Rac1 siRNAs in comparison to scrambled siRNA (Additional file 1). Our initial assumption was confirmed as CM-induced cell elongation was inhibited in Rac1 siRNA-transfected microglia (Fig. 1a, b). The quantification shows that CM-treated microglia that were previously transfected with three individual siRNAs targeting Rac1 or a pool of them have a higher value for circularity, corresponding to a rounder cell shape, compared to control CM-treated microglia that were not transfected or transfected with scrambled siRNAs (Fig. 1c). Thus, these experiments show that our assay is functional and that Rac1 siRNA could be used as specific positive control in this siRNA screen.

### siRNA screening in CM-treated microglia identifies various potential targets to regulate microglial activation

Using this experimental strategy, we next evaluated the effect on CM-induced microglia elongation of a custom-made Silencer-Select siRNA library against 157 mouse proteins including 100 cytoskeletal proteins and 57 regulators of microglia activation/inflammatory pathways. We selected 22 small RhoGTPases [20] and 78 of their activators, Rho guanine nucleotide exchange factors RhoGEFs [21], because they are involved in regulation of cytoskeletal architecture and changes in the cell morphology are intrinsically related to the cytoskeleton. On the other hand, we selected major regulators of inflammatory pathways, including various members of the mitogen-activated protein kinases (MAPK), phosphatidylinositol-4,5-bisphosphate 3-kinase (PI3K) and nuclear factor kappa B subunit 1 (NF-κB) signaling routes, because these proteins are likely to interfere with the CM-induced anti-inflammatory phenotype of microglia and hence have a higher probability to influence the inflammatory state of microglia. The supplementary Tables S1 and S2 in Additional files 2 and 3, respectively, show a list of all genes that were targeted by an initial screening using our siRNA library (a pool of three individual siRNAs) including their effects on the differential circularity. A positive differential circularity (Additional file 2) signifies that the cells were rounder than cells transfected with Scrambled siRNA, and negative values of circularity (Additional file 3) imply that microglia transfected with this gene showed an enhanced ramification pattern. Since we were interested in genes that inhibited the neuroprotective ramified phenotype of microglia, the statistically significant positive hits (genes marked in bold in Additional file 2) were compared to publicly available datasets of PD-associated gene expression [22] and pathways [23]. We also compared them to differential transcriptome expression data from post mortem tissue of the substantia nigra between healthy and PD patients [24]. Overlapping genes between our siRNA screen and those databases as well as the seven hits with the highest differential circularities were subjected to a literature search to collect information about their potential physiological role in microglia. From there, the selected hits Asef (Arhgef4), Tiam1, GM-CSFR (Csf2rA), IκBα (Nfκbia), RhoE (Rnd3), p38β (Mapk11), Creb1, MEK3 (Map2k3), Map3k2, and JNK2 (Mapk9) were then validated in the following step.

**Fig. 2:**
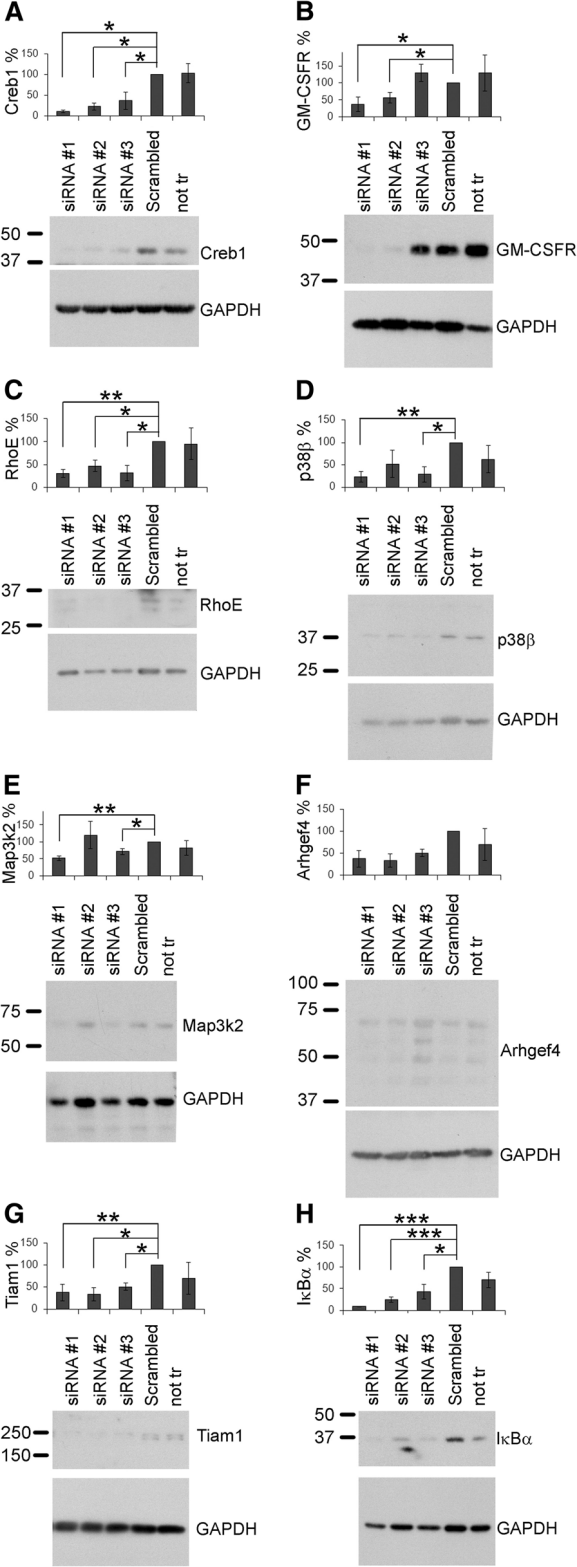
Knockdown validation by Western blot of the positive hits. Primary mouse microglia were transfected with three individual siRNAs against the indicated target genes. After 3 (**b** and **e**) or 4 days (**a**, **c**, **d**, **f**, **g**, and **h**), cells were harvested and subjected to Western blot analysis. Expression of the indicated proteins was normalized to GAPDH expression. Representative Western blots for each protein are depicted below the corresponding graph. Data are mean ± SEM of at least three independent experiments. *p < 0.05, **p < 0.01, ***p < 0.001 vs. Scrambled siRNA

Since siRNA screens in mammalian cells might be influenced by off-target effects [25], we confirmed selected positive hits by transfecting individually the three single siRNAs of the original pool. To exclude these off-target effects, at least two siRNA oligonucleotides targeted against the same gene should exhibit the same phenotypic effect on CM-treated microglia (assayed by changes in circularity values) and efficiency to knock down its gene target (assayed by Western blot analysis). As shown in Table 1, not all positive hits of the previous siRNA screening were verified in this step. None of the three siRNAs against MEK3, and the two siRNAs against JNK2, were able to individually change circularity values in CM-treated microglia (Table 1), and thus, the genes MEK3 and JNK2 were marked as false positives. In addition, the protein levels of all tested genes, except Arhgef4, were significantly downregulated by at least two single siRNAs (Fig. 2). Since none of the individual siRNAs targeting Arhgef4 were able to downregulate the protein Arhgef4, this hit had to be considered as false positive.

**Table 1:** Validation of siRNA targets resulting in positive differential circularity

siRNA	Differential circularity	*P* value	FDR
*Tiam1 #1*	*2.73*	*< 10* ^*−16*^	*< 10* ^*−14*^
*Creb1 #2*	*2.55*	*< 10* ^*−14*^	*< 10* ^*−11*^
*p38β #3*	*2.22*	*< 10* ^*−11*^	*< 10* ^*−10*^
*Tiam1 #2*	*2.06*	*< 10* ^*−10*^	*< 10* ^*−9*^
*RhoE #1*	*1.93*	*< 10* ^*−9*^	*< 10* ^*−8*^
*Arhgef4 #2*	*1.84*	*< 10* ^*−8*^	*< 10* ^*−7*^
*p38β #2*	*1.79*	*< 10* ^*−8*^	*< 10* ^*−7*^
*Creb1 #1*	*1.71*	*< 10* ^*−7*^	*< 10* ^*−6*^
*Map3k2 #1*	*1.64*	*< 10* ^*−6*^	*< 10* ^*−6*^
*Map3k2 #3*	*1.52*	*< 10* ^*−6*^	*< 10* ^*−5*^
*IκBα #2*	*1.46*	*< 10* ^*−5*^	*< 10* ^*−5*^
*GM-CSFR #1*	*1.44*	*< 10* ^*−5*^	*< 10* ^*−5*^
*IκBα #1*	*1.40*	*< 10* ^*−5*^	*< 10* ^*−4*^
*Map3k2 #2*	*1.36*	*< 10* ^*−5*^	*< 10* ^*−4*^
*Tiam1 #3*	*1.26*	*< 10* ^*−4*^	*< 10* ^*−4*^
*p38β #3*	*1.19*	*< 10* ^*−4*^	*< 0.01*
*RhoE #2*	*1.15*	*< 0.001*	*< 0.01*
*JNK2 #3*	*1.08*	*< 0.001*	*< 0.01*
*Creb1 #3*	*1.01*	*< 0.001*	*< 0.01*
*Arhgef4 #1*	*0.90*	*< 0.01*	*< 0.01*
*GM-CSFR #2*	*0.83*	*< 0.01*	*< 0.01*
*RhoE #3*	*0.73*	*< 0.05*	*< 0.05*
MEK3 #2	0.58	0.05	0.07
Arhgef4 #3	− 0.55	0.06	0.08
GM-CSFR #3	0.42	0.16	0.21
MEK3 #3	− 0.32	0.28	0.35
IκBα #3	0.28	0.34	0.40
MEK3 #1	0.16	0.58	0.64
JNK2 #1	− 0.11	0.70	0.75
JNK2 #2	− 0.08	0.80	0.82

Primary microglia were transfected with three single siRNA oligonucleotides (#1, #2, and #3) per gene and tested for their effect on CM-induced microglia ramification. The change in cell morphology was expressed as differential circularity. Results were sorted by their FDR, and statistically significant genes are marked in italics

### Selected hits differentially regulate BDNF production and migration in microglia

**Fig. 3:**
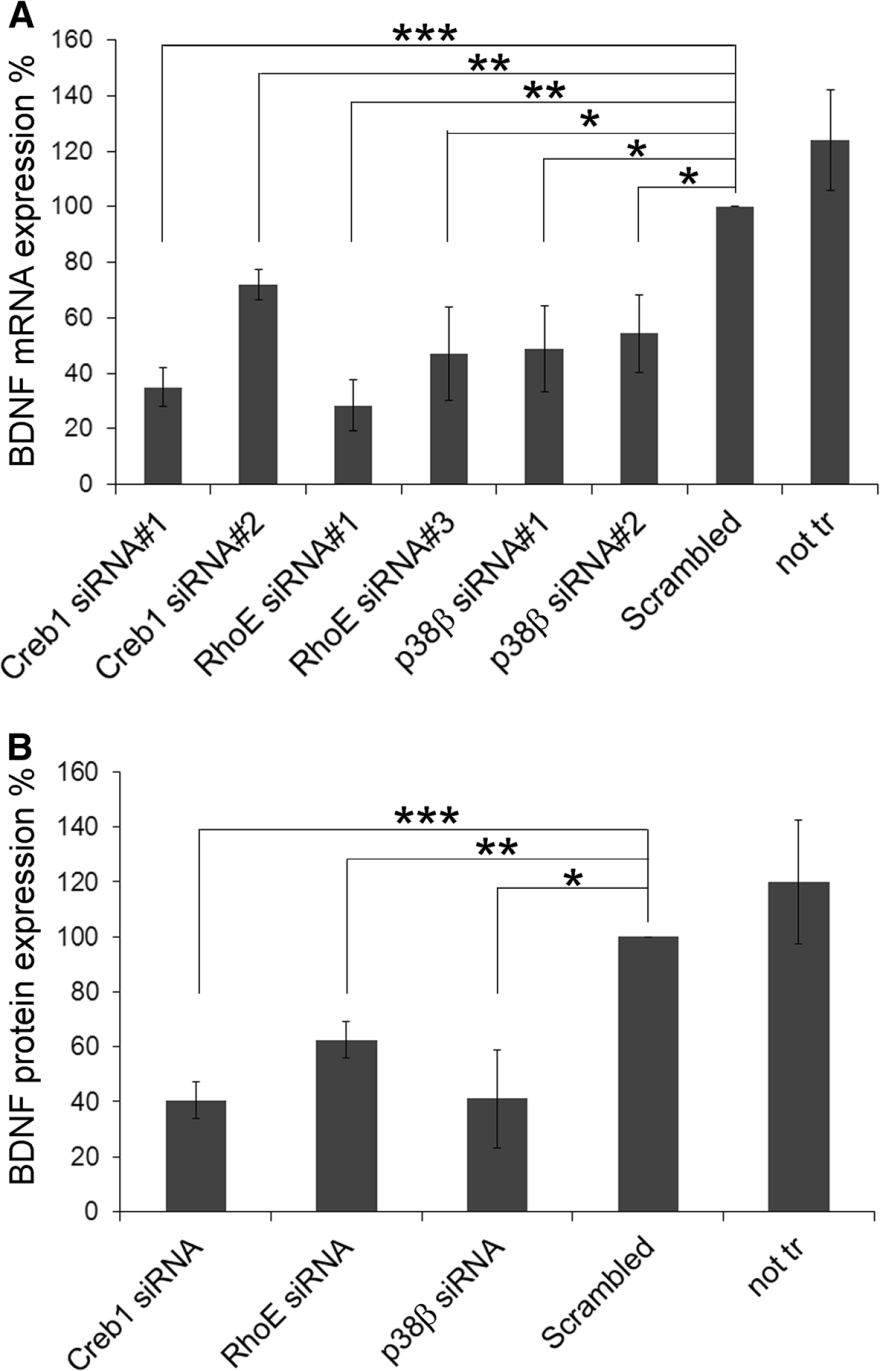
Downregulation of Creb1, RhoE, and p38β reduced BDNF expression in microglia. **a** Primary microglia were transfected with two individual siRNA oligonucleotides against the indicated target genes. Non-transfected cells (not tr) or cells transfected with Scrambled siRNA were used as controls. After 4 days, gene expression of BDNF was determined by RT-qPCR and normalized to GAPDH expression. Data are expressed as BDNF expression relative to that observed in cells transfected with Scrambled siRNA. **b** Primary microglia were transfected with siRNA targeting Creb1 (siRNA#2), RhoE (siRNA#1), and p38β (siRNA#2), as representative siRNAs for these genes. Non-transfected cells (not tr) or cells transfected with Scrambled siRNA were used as controls. After 4 days, culture supernatants were collected and used to determine total BDNF protein expression by ELISA. Data are expressed as BDNF protein expression levels relative to that observed in cells transfected with Scrambled siRNA. Data are mean ± SEM of at least three independent experiments.*p < 0.05, **p < 0.01, ***p < 0.001 vs. Scrambled siRNA

To pinpoint the functional consequences of the ramification impairment, we ran secondary screens with the seven validated hits (Tiam1, GM-CSFR, IκBα, RhoE, p38β, Creb1, and Map3k2) by investigating their effects in two processes that are related to microglia activation or with mechanisms through which microglia might exert their neuroprotective functions, namely cell migration capacity and production of neurotrophic factors. Since CM-induced BDNF production in primary microglia [7], we transfected primary microglia with the two individual siRNAs of the seven positive hits that efficiently downregulated their target protein (see Fig. 2), and measured the expression of BDNF mRNA 4 days later. siRNAs against Creb1, RhoE, and p38β were able to downregulate basal BDNF mRNA expression by microglia (Fig. 3a), suggesting that they could achieve their neuroprotective function via BDNF production. However, siRNAs against Tiam1, GM-CSFR, IκBα, and Map3k2 failed to regulate BDNF mRNA expression (data not shown). To confirm the downregulation of BDNF mRNA at the protein level, we performed an ELISA to measure the total amount of BDNF protein in the culture supernatants of Creb1, RhoE, and p38β siRNA-transfected cells. Indeed, downregulation of these three proteins also resulted in a significant reduction of BDNF protein (Fig. 3b).

**Fig. 4:**
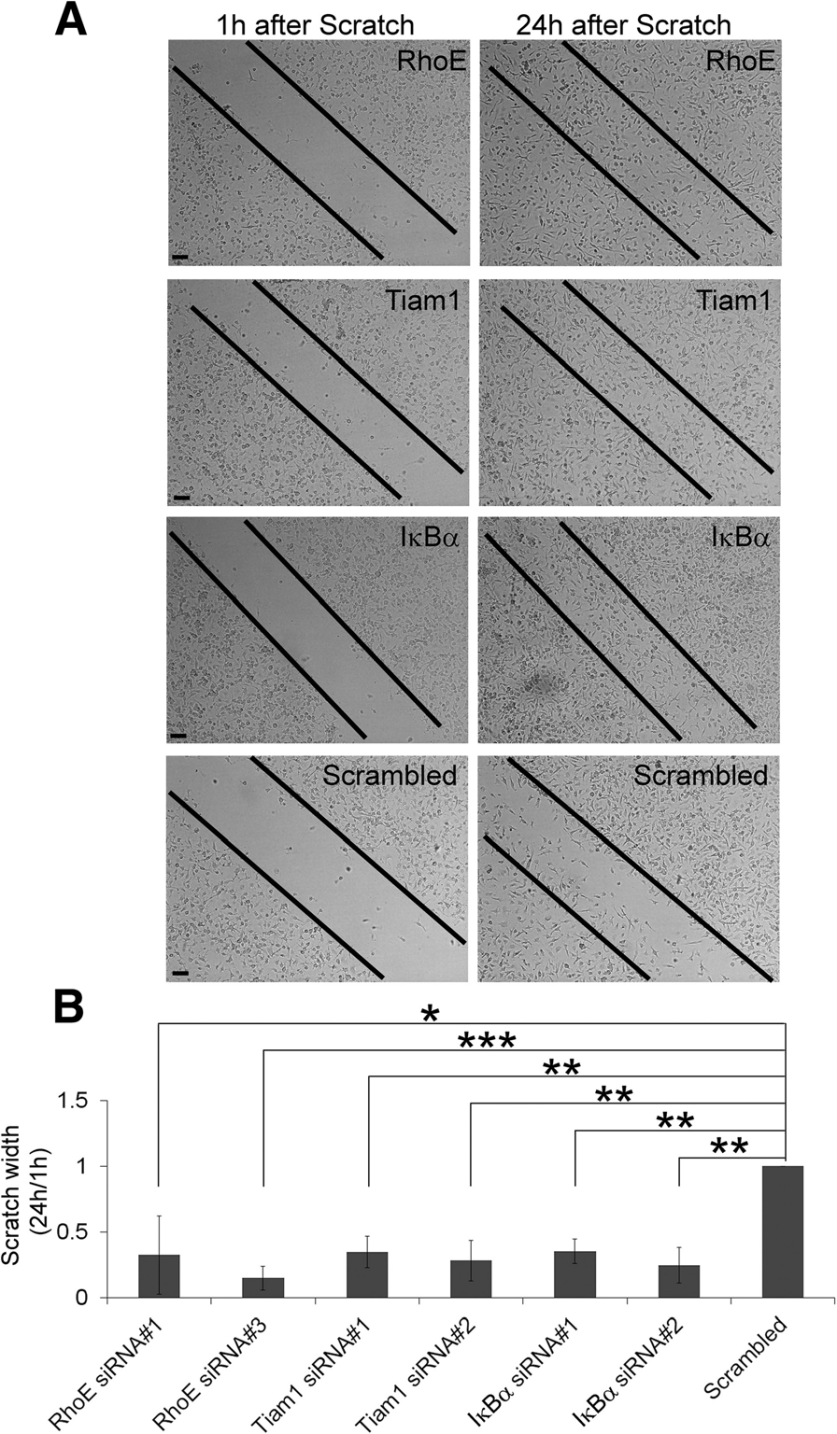
Downregulation of RhoE, Tiam1, and IκBα enhanced microglia migration. Primary microglia were transfected with two individual siRNA oligonucleotides against the indicated target genes for 3 days and then treated with CM. Scrambled siRNA was used as control. Cell migration was determined in a 24-h scratch assay. **a** Representative images of the scratch width at 1 and 24 h are shown. **b** The scratch width at 24 h was determined as a percentage of the original scratch width at 1 h and then expressed relative to the scratch width of the Scrambled siRNA-transfected cells (normalized to 1.0) in each experiment. Data are mean ± SEM of at least three independent experiments.*p < 0.05, **p < 0.01, ***p < 0.001 vs. Scrambled siRNA

On the other hand, since the amoeboid cell shape in microglia is associated with cell migration [26, 27], we used a scratch assay to test the effect of our seven validated hits on microglia migration. We transfected primary microglia with two individual siRNAs of our positive hits, and 3 days later, we added CM and scratched the cover slip. We observed that microglia transfected with siRNAs against RhoE, Tiam1, or IκBα siRNAs closed their scratch prior to Scrambled siRNA (Fig. 4), indicating that these three genes are negative regulators for microglia migration. In contrast, siRNAs against GM-CSFR, p38β, Creb1, and Map3k2 did not affect their migratory capacity (data not shown). The downregulation of RhoE, Tiam1, or IκBα by siRNA transfection did not affect MTT reduction in microglia cultures (data not shown), suggesting that their effects measured by the scratch assay are not due to an action on cell viability/proliferation. Table 2 summarizes the results of both secondary screens.

**Table 2:** Summary of the secondary screens

Validated positive hit	BDNF production	Microglia migration
RhoE/Rnd3	**Reduced**, ***p*<0.01**	**Enhanced**, ***p*<0.01**
Tiam1	Not affected	**Enhanced**, ***p*<0.01**
p38β/Mapk11	**Reduced**, ***p*<0.05**	Not affected
GM-CSFR/Csf2ra	Not affected	Not affected
Map3k2	Not affected	Not affected
Creb1	**Reduced**, ***p*<0.001**	Not affected
IκBα/Nfkbia	Not affected	**Enhanced**, ***p*<0.01**

Primary microglia were transfected with validated siRNA oligonucleotides against their indicated target protein and tested for their effect on BDNF expression and microglia migration as described in Figs. 3 and 4

## Discussion

Highly elongated and ramified microglia are predominantly found in a healthy brain [8]. But it is commonly accepted that microglia can display an amoeboid morphology during a neuroinflammatory response [6], for example, as seen in PD [4]. Although microglia ramification is equally important as the development of axons and dendrites for neurons to exert their functions, very little is known about the pathways that induce this ramification. To systematically unravel this process, we performed a microscopy-based siRNA screen in primary microglia and have identified a list of 45 genes that are positively involved in the ramification of microglia. Since we are aware of high numbers of false positive hits in siRNA screens [28], we have applied a rigorous validation procedure in our siRNA screen in order to make our hits reliable and reproducible results. The execution of this validation step seems to be essential, because we observed that three of ten initially selected positive hits were false positives.

Two of the seven positive hits, the Rho guanine nucleotide exchange factor (RhoGEF) Tiam1 and the small RhoGTPase RhoE (also known as Rnd3) are regulators of the cytoskeleton. The NF-κB inhibitor IκBα, the kinases p38β (also known as Mapk11) and Map3k2, the transcription factor Creb1, and the cytokine receptor for granulocyte macrophage colony stimulating factor (GM-CSFR, also known as Csf2ra) are molecules involved in inflammatory responses. Since their knock down inhibited microglia ramification upon CM treatment, a potent anti-inflammatory neuroprotective stimulus [7], these seven proteins must play an essential role in this process and their identification sheds the first light on these so far poorly characterized signaling routes.

The fact that the BDNF expression is affected by the downregulation of three out of seven positive hits indicates that this might be a possible mechanism for acquiring a neuroprotective phenotype. Indeed, a decreased release of BDNF by microglia is one of the mechanisms by which microglia can kill neurons [27]. Since BDNF transcription is mediated by Creb1 [29], our result that BDNF mRNA expression was reduced by Creb1 siRNA was actually not surprising. Interestingly, RhoE has been identified as BDNF-induced Creb1-regulated gene in hippocampal neurons [30]. Now, we report here for the first time that RhoE vice-versa also regulates BDNF expression, establishing a potential positive feedback similar to that previously described for Creb1 activation-BDNF expression [29]. Because this positive feedback-loop is one of the reasons to consider to Creb1 as a key player in neuronal survival [31], our new findings with RhoE could result of relevance from a physiological point of view.

The MAPK p38 is expressed as two major isoforms in the brain, namely p38α and p38β, which have around 70% homology. Most of the published work so far refers to p38α and only few data is available distinguishing both isoforms, mainly because the main p38 inhibitors target both isoforms [32]. However, a recent report showed that siRNA-mediated downregulation of p38β, but not of p38α, resulted in impaired Creb1 activation in microglia [33]. These results are in good concordance with our results showing that p38α (Mapk14, Additional file 3) did not come up as positive hit in our primary screen for deactivating-microglia drug targets. Thus, these findings suggest a differential role of both isoforms of p38 in microglia activity states, with p38β mainly being implicated in a neuroprotective phenotype.

Besides affecting particular changes in cell morphology and gene expression, positive hits also changed microglia migration, being a more global cellular read-out. Migration is one of the important functions of microglia to assess a lesion site once injury or insult in the brain has occurred and is typically associated with an inflammatory phenotype [27]. Three of our positive hits, namely Tiam1, RhoE, and IκBα, enhanced cell migration when downregulated by siRNA. It was previously described that downregulation of RhoE promoted glioblastoma cell migration and invasion [34]. On the other hand, although the RhoGEF Tiam1 is traditionally believed to enhance cell migration, its role in cell adhesion makes its involvement in cell migration controversial depending on cell type and extracellular matrix used [35]. Under our experimental conditions, Tiam1 would be rather involved in cell adhesion, since its downregulation enhances primary microglia migration. Regarding IκBα, it is likely that it exerts its neuroprotective role in microglia by inhibiting the inflammatory signaling of the transcription factor NF-κB, probably by retaining it in the cytoplasm [36]. The role of IκBα itself in cell migration is less studied, but for example curcumin suppresses inflammatory-induced NF-κB signaling and macrophage migration [37]. In addition, pharmacological inhibition of nuclear translocation of NF-κB blocked endothelial cell migration in a scratch assay [38]. These results correlate with ours, namely that the downregulation of IκBα, which probably increases nuclear NF-κB, enhanced microglia migration.

Since in our secondary screens not all hits overlap, we must assume that our seven validated hits probably do not act on the same pathway, probably because the CM we applied is a mix of several growth factors and cytokines and can stimulate several pathways at once. It is however remarking that RhoE came up as hit in both analyses, placing RhoE in a central role to act on microglia activation states. RhoE is an atypical RhoGTPase, because its GTPase domain is not able to hydrolyze GTP, being its activity regulated by its expression level and phosphorylation [39]. As mentioned above, its gene expression can be regulated by BDNF [30]. RhoE’s main function is to act as endogenous antagonist to RhoA-mediated actin cytoskeleton remodeling, which is especially important in cell migration and polarization. For example, polarization and axonal and dendritic lengths are reduced in RhoE-null neurons [40], a comparable phenotype to that we observed in RhoE-downregulated microglia, which were less ramified. Taken all these data together, we suggest RhoE is a promising candidate gene, whose action is required for the reversion of microglia into a neuroprotective phenotype, being especially indicative its role in BDNF production. However, further studies are necessary to establish its precise molecular role in this process. Since RhoE, similar to p38β, has not been previously identified publicly available databases related to PD, these findings are completely novel in the context of neurodegenerative diseases.

Finally, our primary siRNA screen also identified 50 genes (Additional file 3) that potentially block the acquisition of a ramified morphology in microglia. This suggests that selective inhibitors against these molecules would favor this neuroprotective phenotype. Although the validation of these negative hits is out of the scope of the present study, it offers this possibility to other researchers.

## Conclusions

In summary, with the first siRNA screen performed in primary microglia, we generated a validated database of intracellular molecules that play a role in the transition of microglia from an activated damaging phenotype to a potential neuroprotective one. Hence, our hits might represent possible novel drug targets in neurodegenerative diseases with an underlying neuroinflammatory process. With these important elementary results, we open up new investigation lines to reveal the mechanism involved in this process and generate knowledge in order to discover innovative treatments against neurodegenerative disorders by preventing neuronal death mediated by over-activated microglia.

## Additional files

Additional file 1: Rac1 siRNA transfection efficiently downregulated Rac1 protein and gene in microglia. (A) Primary microglia (140,000 cells/well, 12-well plates) were transfected with 50 pmol Rac1 of Silencer Select Rac1 siRNA or Scrambled siRNA (used as control) and 1.5 μl Lipofectamine 3000. Rac1 protein expression was determined by Western blot analysis 96 h later. (B) and (C) Primary microglia were transfected as in (A) for the indicated time periods, and Rac1 downregulation was evaluated by RT-qPCR (B) and by Western blot (C). Data are mean ± SEM of three independent experiments.**p < 0.01, ***p < 0.001* vs. Scrambled siRNA. (JPG 301 kb)

Additional file 2: **Table S1.** List of siRNA targets resulting in positive differential circularity. Primary microglia were transfected with a pool of siRNA oligonucleotides against each gene and tested for their effect on CM-induced microglia ramification. The result was expressed as differential circularity. siRNAs that caused positive differential circularity values were selected and sorted by their fold discovery rate (FDR), which represents an adjusted *P* value. Statistically significant genes were marked in bold. Genes that were annotated by the Parkinson’s UK Gene Ontology Project [22] are marked in the PD gene column. Genes that were listed in the PD map tool of the University of Luxembourg (http://pdmap.uni.lu/minerva/) are marked in the PD map column [23]. Genes overlapping with differential transcriptome expression data from post mortem tissue of the substantia nigra [24] published on the PD map webpage (http://pdmap.uni.lu/minerva/) are marked in the last column. *Positive hits that were selected for validation. (XLSX 55 kb)

Additional file 3: **Table S2.** List of siRNA targets resulting in negative differential circularity. Primary microglia were transfected with a pool of siRNA oligonucleotides against each gene and tested for their effect on CM-induced microglia ramification, which was expressed as differential circularity. siRNAs that resulted in negative differential circularity values were selected and sorted by their fold discovery rate (FDR). Statistically significant genes were marked in bold. Genes that were annotated by the Parkinson’s UK Gene Ontology Project [22] are marked in the PD genes column. Genes that were listed in the PD map tool of the University of Luxembourg (http://pdmap.uni.lu/minerva/) are marked in the PD map column [23]. Genes overlapping with differential transcriptome expression data from post mortem tissue of the substantia nigra [24] published on the PD map webpage (http://pdmap.uni.lu/minerva/) are marked in the last column. (XLSX 56 kb)

## Abbreviations

ASCs: Adipose-derived mesenchymal stem cells
BDNF: Brain-derived neurotrophic factor
CM: Conditioned medium of ASCs
CNS: Central nervous system
FDR: Fold discovery rate
GM-CSFR: Receptor for granulocyte macrophage colony stimulating factor
IκBα: Inhibitor of NF-κB
MAPK: Mitogen-activated protein kinases
NF-κB: Nuclear factor kappa B subunit 1
PD: Parkinson’s disease
PI3K: Phosphatidylinositol-4,5-bisphosphate 3-kinase
RhoGEF: Rho guanine nucleotide exchange factor
siRNA: Small interference RNA
Tiam1: T-lymphoma invasion and metastasis-inducing protein 1

## References

[1] Qian L, Flood PM, Hong JS (2010). Neuroinflammation is a key player in Parkinson’s disease and a prime target for therapy. J Neural Transm.

[2] Gonzalez H, Elgueta D, Montoya A, Pacheco R (2014). Neuroimmune regulation of microglial activity involved in neuroinflammation and neurodegenerative diseases. J Neuroimmunol.

[3] Wolf SA, Boddeke HW, Kettenmann H (2017). Microglia in physiology and disease. Annu Rev Physiol.

[4] Doorn KJ, Goudriaan A, Blits-Huizinga C, Bol JG, Rozemuller AJ, Hoogland PV (2014). Increased amoeboid microglial density in the olfactory bulb of Parkinson’s and Alzheimer’s patients. Brain Pathol.

[5] Block ML, Zecca L, Hong JS (2007). Microglia-mediated neurotoxicity: uncovering the molecular mechanisms. Nat Rev Neurosci.

[6] Kettenmann H, Hanisch UK, Noda M, Verkhratsky A (2011). Physiology of microglia. Physiol Rev.

[7] Neubrand VE, Pedreno M, Caro M, Forte-Lago I, Delgado M, Gonzalez-Rey E (2014). Mesenchymal stem cells induce the ramification of microglia via the small RhoGTPases Cdc42 and Rac1. Glia.

[8] Hanisch UK, Kettenmann H (2007). Microglia: active sensor and versatile effector cells in the normal and pathologic brain. Nat Neurosci.

[9] Nobes CD, Hall A (1995). Rho, rac, and cdc42 GTPases regulate the assembly of multimolecular focal complexes associated with actin stress fibers, lamellipodia, and filopodia. Cell.

[10] Bishop AL, Hall A (2000). Rho GTPases and their effector proteins. Biochem J.

[11] Krausz E (2007). High-content siRNA screening. Mol BioSyst.

[12] Demir K, Boutros M (2012). Cell perturbation screens for target identification by RNAi. Methods Mol Biol.

[13] Horvath RJ, Nutile-McMenemy N, Alkaitis MS, Deleo JA (2008). Differential migration, LPS-induced cytokine, chemokine, and NO expression in immortalized BV-2 and HAPI cell lines and primary microglial cultures. J Neurochem.

[14] Kilkenny C, Browne W, Cuthill IC, Emerson M, Altman DG, Group NCRRGW (2010). Animal research: reporting in vivo experiments: the ARRIVE guidelines. Br J Pharmacol.

[15] Anderson P, Souza-Moreira L, Morell M, Caro M, O'Valle F, Gonzalez-Rey E (2013). Adipose-derived mesenchymal stromal cells induce immunomodulatory macrophages which protect from experimental colitis and sepsis. Gut.

[16] Wilms H, Hartmann D, Sievers J (1997). Ramification of microglia, monocytes and macrophages in vitro: influences of various epithelial and mesenchymal cells and their conditioned media. Cell Tissue Res.

[17] Boutros M, Bras LP, Huber W (2006). Analysis of cell-based RNAi screens. Genome Biol.

[18] Smyth GK (2004). Linear models and empirical bayes methods for assessing differential expression in microarray experiments. Stat Appl Genet Mol Biol.

[19] Benjamini Y, Hochberg Y (1995). Controlling the false discovery rate: a practical and powerful approach to multiple testing. J R Stat Soc Ser B.

[20] Bustelo XR, Sauzeau V, Berenjeno IM (2007). GTP-binding proteins of the Rho/Rac family: regulation, effectors and functions in vivo. BioEssays.

[21] Rossman KL, Der CJ, Sondek J (2005). GEF means go: turning on RHO GTPases with guanine nucleotide-exchange factors. Nat Rev Mol Cell Biol.

[22] Foulger RE, Denny P, Hardy J, Martin MJ, Sawford T, Lovering RC (2016). Using the gene ontology to annotate key players in Parkinson’s disease. Neuroinformatics.

[23] Fujita KA, Ostaszewski M, Matsuoka Y, Ghosh S, Glaab E, Trefois C (2014). Integrating pathways of Parkinson’s disease in a molecular interaction map. Mol Neurobiol.

[24] Glaab E, Schneider R (2015). Comparative pathway and network analysis of brain transcriptome changes during adult aging and in Parkinson’s disease. Neurobiol Dis.

[25] Singh S, Narang AS, Mahato RI (2011). Subcellular fate and off-target effects of siRNA, shRNA, and miRNA. Pharm Res.

[26] Jonas RA, Yuan TF, Liang YX, Jonas JB, Tay DK, Ellis-Behnke RG (2012). The spider effect: morphological and orienting classification of microglia in response to stimuli in vivo. PLoS One.

[27] Brown GC, Vilalta A (2015). How microglia kill neurons. Brain Res.

[28] Birmingham A, Selfors LM, Forster T, Wrobel D, Kennedy CJ, Shanks E (2009). Statistical methods for analysis of high-throughput RNA interference screens. Nat Methods.

[29] West AE, Griffith EC, Greenberg ME (2002). Regulation of transcription factors by neuronal activity. Nat Rev Neurosci.

[30] Lesiak A, Pelz C, Ando H, Zhu M, Davare M, Lambert TJ (2013). A genome-wide screen of CREB occupancy identifies the RhoA inhibitors Par6C and Rnd3 as regulators of BDNF-induced synaptogenesis. PLoS One.

[31] Walton MR, Dragunow I (2000). Is CREB a key to neuronal survival?. Trends Neurosci.

[32] Yasuda S, Sugiura H, Tanaka H, Takigami S, Yamagata K (2011). p38 MAP kinase inhibitors as potential therapeutic drugs for neural diseases. Cent Nerv Syst Agents Med Chem.

[33] Wu HY, Mao XF, Fan H, Wang YX (2017). p38beta mitogen-activated protein kinase signaling mediates exenatide-stimulated microglial beta-endorphin expression. Mol Pharmacol.

[34] Liu B, Dong H, Lin X, Yang X, Yue X, Yang J (2016). RND3 promotes Snail 1 protein degradation and inhibits glioblastoma cell migration and invasion. Oncotarget.

[35] Boissier P, Huynh-Do U (2014). The guanine nucleotide exchange factor Tiam1: a Janus-faced molecule in cellular signaling. Cell Signal.

[36] Li Q, Verma IM (2002). NF-kappaB regulation in the immune system. Nat Rev Immunol.

[37] Young NA, Bruss MS, Gardner M, Willis WL, Mo X, Valiente GR (2014). Oral administration of nano-emulsion curcumin in mice suppresses inflammatory-induced NFkB signaling and macrophage migration. PLoS One.

[38] Liu C, Tsai AL, Li PC, Huang CW, Wu CC (2017). Endothelial differentiation of bone marrow mesenchyme stem cells applicable to hypoxia and increased migration through Akt and NFkappaB signals. Stem Cell Res Ther.

[39] Jie W, Andrade KC, Lin X, Yang X, Yue X, Chang J (2015). Pathophysiological functions of Rnd3/RhoE. Compr Physiol.

[40] Peris B, Gonzalez-Granero S, Ballester-Lurbe B, Garcia-Verdugo JM, Perez-Roger I, Guerri C (2012). Neuronal polarization is impaired in mice lacking RhoE expression. J Neurochem.

