# Correction to: The atypical RhoGTPase RhoE/Rnd3 is a key molecule to acquire a neuroprotective phenotype in microglia

**DOI:** 10.1186/s12974-019-1416-5

**Published:** 2019-02-12

**Authors:** Veronika E. Neubrand, Irene Forte-Lago, Marta Caro, Mario Delgado

**Affiliations:** Instituto de Parasitología y Biomedicina López-Neyra, IPBLN-CSIC, Avd. Conocimiento 17, PTS Granada, 18016 Granada, Spain

**Correction to: J Neuroinflammation**

**https://doi.org/10.1186/s12974-018-1386-z**

In the version of this article that was originally published [1]; some information in the “Authors’ contributions” section was omitted. This has now been corrected in this Correction note.
