# Supplementary figures and images for: The atypical RhoGTPase RhoE/Rnd3 is a key molecule to acquire a neuroprotective phenotype in microglia
Source: J Neuroinflammation. 2018 Dec 15;15:343. doi: 10.1186/s12974-018-1386-z (PMC6295018; doi:10.1186/s12974-018-1386-z)

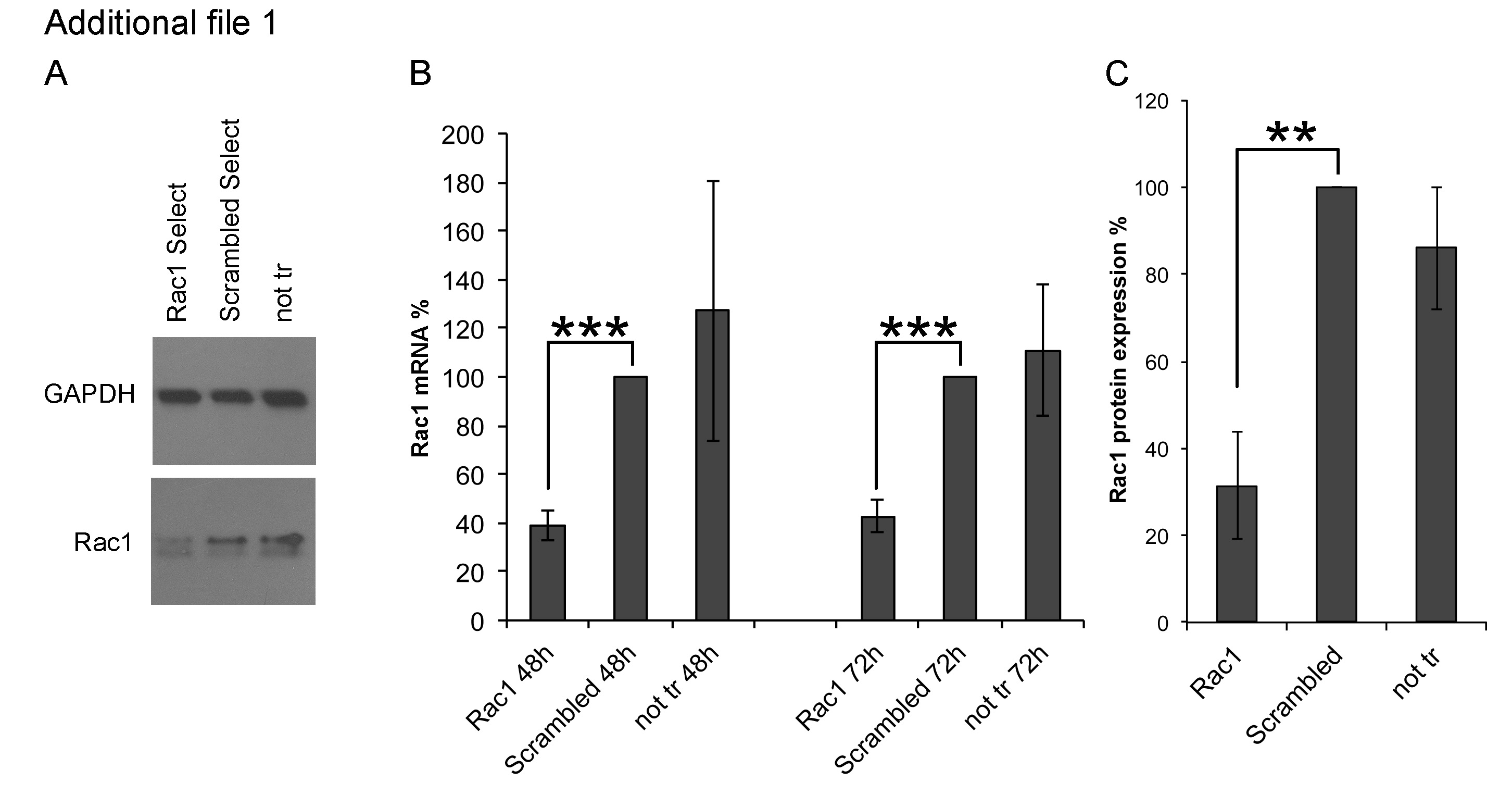

Supplement: Supplementary file 1 — Rac1 siRNA transfection efficiently downregulated Rac1 protein and gene in microglia. (A) Primary microglia (140,000 cells/well, 12-well plates) were transfected with 50 pmol Rac1 of Silencer Select Rac1 siRNA or Scrambled siRNA (used as control) and 1.5 μl Lipofectamine 3000. Rac1 protein expression was determined by Western blot analysis 96 h later. (B) and (C) Primary microglia were transfected as in (A) for the indicated time periods, and Rac1 downregulation was evaluated by RT-qPCR (B) and by Western blot (C). Data are mean ± SEM of three independent experiments.**p < 0.01, ***p < 0.001* vs. Scrambled siRNA. (JPG 301 kb) [file 12974_2018_1386_MOESM1_ESM.jpg]
